# Prognostic Role of Systemic Inflammatory Indexes in Germ Cell Tumors Treated With High-Dose Chemotherapy

**DOI:** 10.3389/fonc.2020.01325

**Published:** 2020-08-14

**Authors:** Maria Concetta Cursano, Barbara Kopf, Emanuela Scarpi, Cecilia Menna, Chiara Casadei, Giuseppe Schepisi, Cristian Lolli, Amelia Altavilla, Valentina Gallà, Daniele Santini, Giuseppe Tonini, Michal Chovanec, Michal Mego, Ugo De Giorgi

**Affiliations:** ^1^Department of Medical Oncology, University of Rome, Rome, Italy; ^2^Department of Medical Oncology, Istituto Scientifico Romagnolo per lo Studio e la Cura dei Tumori (IRST) IRCCS, Meldola, Italy; ^3^Unit of Biostatistics and Clinical Trials, Istituto Scientifico Romagnolo per lo Studio e la Cura dei Tumori (IRST) IRCCS, Meldola, Italy; ^4^Second Department of Oncology, Faculty of Medicine, Comenius University, Bratislava, Slovakia; ^5^Department of Oncology, National Cancer Institute, Bratislava, Slovakia; ^6^Translational Research Unit, Faculty of Medicine, Comenius University, Bratislava, Slovakia

**Keywords:** germ cell tumors, neutrophil-to-lymphocyte ratio, systemic immune-inflammation index, high-dose chemotherapy, prognostic factor, immunity

## Abstract

High-dose chemotherapy (HDCT) has curative potential in relapsed/refractory germ cell tumors (GCT). Due to the complexity of this population and the toxicity of HDCT, we evaluated the association between blood-based systemic inflammatory indexes and the outcome of GCT patients undergoing salvage treatment with HDCT in order to define additional prognostic factors able to orient clinical decision. Baseline characteristics, neutrophil-to-lymphocyte ratio (NLR), platelet-to-lymphocyte ratio (PLR), and the systemic immune-inflammation index (SII) of 62 patients undergoing HDCT for GCT were retrospectively collected. The aim is to evaluate the correlation between each inflammatory marker (NLR, PLR, and SII) and response to HDCT, overall survival (OS), and progression-free survival (PFS). Using the receiver operating curve to identify the best cutoff values, it was found that patients with GCT with NLR ≥3.3 and SII ≥844,000 had shorter PFS and inferior OS. In the multivariable analysis including inflammatory markers, the International Prognostic Factor Study Group (IPFSG) risk group, age, and previous line of treatment, NLR ≥3.3 and SII ≥844,000 were identified to be independently associated with shorter PFS and OS. Moreover, NLR, PLR, and SII significantly correlate with overall response to HDCT. Associating IPFSG prognostic score to inflammatory markers at baseline of HDCT may improve prognostic information and could help physicians to make more personalized treatment decisions.

## Introduction

Metastatic germ cell tumors (GCT) are extremely chemo-sensitive tumors with a rate of relapse of only 20% after cisplatin-based first-line chemotherapy (1, 2). The two salvage curative approaches are conventional-dose chemotherapy (CDCT) and high-dose chemotherapy (HDCT) with support of peripheral blood progenitor cells (PBPC). CDCT can induce long-term remissions in 30–50% (3, 4) as second-line therapy, but its efficacy is inferior in patients with multiple relapsed or cisplatin-refractory GCT (5, 6). HDCT can induce durable remissions in a higher percentage of cases, especially in patients with unfavorable characteristics. In particular, extragonadal GCT and patients with brain metastases are notoriously associated with an inferior survival rate with CDCT (7–9). Since both CDCT and HDCT have shown a curative potential in the management of relapsed/refractory GCT, great efforts have been made in order to define the prognostic factors able to orient clinical decision. The International Prognostic Factor Study Group (IPFSG) developed a prognostic model for relapsed/refractory GCT planned for initial salvage chemotherapy (10). In recent years, several studies have shown the prognostic and the predictive role of inflammation indexes such as neutrophil-to-lymphocyte ratio (NLR), platelet-to-lymphocyte ratio (PLR), and systemic immune-inflammation index (SII) in solid tumors (11–15).

In this retrospective analysis, we evaluated the prognostic implication of baseline NLR, PLR, and SII in patients who underwent salvage HDCT with PBPC support. We also aimed to understand if inflammatory markers could add prognostic information to the well-established IPFSG prognostic score.

## Materials and Methods

This is a retrospective study of a consecutive series of GCT patients treated with HDCT and PBPC reinfusion at the Department of Medical Oncology, Istituto Scientifico Romagnolo per lo Studio e la Cura dei Tumori (IRST) IRCCS, Meldola, Italy, from August 2009 to April 2018. All the patients included in the study had histologically or tumor marker-proven relapsed GCT and were candidates for salvage therapy with HDCT. Patient characteristics including age, sex, histology, metastatic sites, prior treatments received, risk group according to IPFSG criteria, blood tests and tumor markers before starting HDCT, data about mobilization chemotherapy regimen, and response to HDCT were collected retrospectively. Information on neutrophil, lymphocyte, and platelet counts from blood tests carried out at baseline (before HDCT treatment) was collected. In addition, the NLR, PLR, and SII were calculated prior to the beginning of HDCT. SII was calculated as platelet count (cells/mm^3^) × neutrophil count (cells/mm^3^)/lymphocyte count (cells/mm^3^), NLR was obtained by dividing the absolute neutrophil count (cells/mm^3^) by the absolute lymphocyte count (cells/mm^3^), and PLR was calculated as the ratio of absolute platelet count (cells/mm^3^) to absolute lymphocyte count (cells/mm^3^). All the patients gave informed consent for the data collection. The local ethics committee approved the study. HDCT was constituted by a first phase of mobilization of PBPC with granulocyte-colony-stimulating factor (G-CSF) and chemotherapy and a second phase of three cycles of HDCT with carboplatin [area under the curve (AUC), 7–8] and etoposide (400 mg/m^2^) on days 1–3, repeated every 21 days, followed by reinfusion of PBPC (16). The chemotherapy mobilizing regimens were paclitaxel and ifosfamide; cisplatin, etoposide, and ifosfamide; cisplatin, bleomicin, and etoposide; and paclitaxel, ifosfamide, and cisplatin. The mobilization chemotherapy regimen was under medical discretion, depending on the previous treatments performed by the single patient. We used the WHO definitions for response criteria for GCTs as follows: Progressive disease (PD) was defined as an increment superior to 25% in bidimensional tumor measurements or by increasing tumor markers of more than 10%, or the appearance of new lesions. Stable disease (SD) was stated as the presence of reduction inferior to 50% or increase inferior to 25% in bidimensional tumor measurements or stable tumor markers. A partial response (PR) was defined as >50% decrease in bidimensional tumor measurements, with >90% reduction in tumor markers. Moreover, the patients who presented no normalized tumor markers with a reduction >90% from baseline were classified as marker-positive partial responders (PRm+), whereas the marker-negative partial responders (PRm–) were those patients with PR and tumor marker normalization. A complete response (CR) to chemotherapy was defined as the disappearance of all clinical, radiographic, and biochemical evidences of disease for at least 4 weeks. Complete response includes the following indicators: patients with complete disappearance of disease after HDCT without residual masses (clinical complete remission, cCR), patients underwent to surgical resection of residual disease and finding of necrosis, fibrosis, or mature teratoma, without evidence of viable malignant cells in the histological report (pathological complete response, pCR), and patients with complete response to chemotherapy plus surgery (sCR, defined as the complete excision of all masses, at least one of which contained viable GCT, other than mature teratoma).

### Statistical Analysis

We performed a univariate Cox regression analysis to assess the association of each systemic inflammatory markers with OS and PFS. Multivariable Cox regression analyses were performed including IPFSG risk groups, age, line of treatment, and each individual systemic inflammatory marker. Systemic inflammatory markers in univariate and multivariable analyses were evaluated as dicotomic variables and were compared using chi-squared or Fisher test as appropriate. We identified an optimal cutoff value for each marker using the receiver operating characteristic (ROC) curve. Time-dependent ROC curves were produced in order to establish the cutoff between low and high expression of baseline NLR, PLR, and SII that yielded the most accurate prediction of PFS and OS at 24 months. Statistical analyses were performed using SAS statistical software version 9.4 (SAS Institute Inc., Cary, NC, USA). Continuous non-normally distributed variables are presented as median and interquartile ranges, and categorical variables are presented as percentage. All *P* < 0.05 were considered as statistically significant. All statistical tests were two-sided. The OS was defined as the period from the date of the beginning of HDCT to the date of death for any causes (extrapolated from death certificates or medical record) or to the date of the last follow-up. The PFS was calculated from the date of the beginning of HDCT to the date of radiological or tumor marker progression or last tumor evaluation.

## Results

Sixty-two patients received HDCT with PBPC infusion and were eligible to be included in this retrospective analysis. Sixty patients (96.8%) were male, 51 (82.3%) with non-seminomatous and nine (14.5%) with seminoma GCT. The remaining two patients were female with a diagnosis of ovarian dysgerminoma. The majority of patients (*n* = 38, 61.3%) received HDCT in second-line setting, while 20 (32.3%) were in third-line setting and four (6.4%) patients were beyond third-line setting. Twenty-five patients (40.3%) and 13 (21%) were classified as IPFSG high and very high risk, respectively. Seventeen patients (27.4%) were classified as intermediate IPFSG risk, six patients (9.7%) were classified as low IPFSG risk, and one patient (1.6%) was classified as very low IPFSG risk (Table 1).

**Table 1:** Patients' baseline characteristics.

**Characteristic**	**Number of patients (%) *n* = 62**
Age, years (median, range)	33 (18–52)
**Sex**	
- Male	60 (96.8)
- Female	2 (3.2)
**Tumor primary site**	
- Gonadal	46 (74.2)
- Mediastinal	8 (12.9)
- Retroperitoneal	7 (11.3)
- Central nervous system	1 (1.6)
**Histology**	
- Pure seminoma	9 (14.5)
- Non-seminoma or mixed	51 (82.3)
- Ovarian dysgerminoma	2 (3.2)
**Transplant setting**	
- Second line	38 (61.3)
- Third line	20 (32.3)
- Beyond third line	4 (6.4)
**Bone, liver, or brain metastasis prior to HDCT**	
- Yes	23 (37.1)
- No	39 (62.9)
**Serum AFP (ng/ml) prior to HDCT**	
- Normal AFP level	36 (58.1)
- Serum AFP level <10,000	25 (40.3)
- Serum AFP level ≥10,000	1 (1.6)
**Serum** **β-human chorionic gonadotropin (hCG, mUI/ml) prior to HDCT**	
- Normal hCG level	31 (50)
- Serum hCG level <50,000	29 (46.8)
- Serum hCG level ≥50,000	2 (3.2)
**Serum LDH prior to HDCT**	
- LDH <1.5 × ULN	49 (79)
- LDH >1.5 and <10 × ULN	12 (19.4)
- LDH >10 × ULN	1 (1.6)
**Risk per IPFSG criteria**	
- Very low	1 (1.6)
- Low	6 (9.7)
- Intermediate	17 (27.4)
- High	25 (40.3)
- Very high	13 (17)
**Mobilization chemotherapy**	
- Ifosfamide paclitaxel	37 (59.7)
- PEI	13 (17)
- TIP	7 (11.3)
- PEB	4 (6.4)
- PE	1 (1.6)
**Chemotherapy mobilization cycles**	
- Median (range)	1 (1–3)
**Carboplatin etoposide (HDCT) received**	
−0 cycle	6 (9.6)
−1 cycle	4 (6.5)
−2 cycles	4 (6.5)
−3 cycles	48 (77.4)

*PEI, cisplatin, etoposide, and ifosfamide; PEB, cisplatin, etoposide, and bleomicin; TIP, paclitaxel, ifosfamide, and cisplatin; PE, cisplatin and etoposide; HDCT, high-dose chemotherapy; LDH, lactate dehydrogenase; ULN, upper limit of normal; hCG, human chorionic gonadotropin; AFP, alpha-fetoprotein; IPFSG, International Prognostic Factors Study Group*.

The median follow-up was 68 months (range, 4–110 months). Every patient underwent a mobilization schedule with chemotherapy and G-CSF. The patients underwent in median 1 cycle (range, 1–3) of the mobilization chemotherapy as necessary to achieve an adequate number of peripheral blood stem cells. The cutoff value derived from ROC curves analysis was 3.3 (AUC 67.7%, 95% CI 52.8–82.6) for NLR, 170 (AUC 54.1%, 95% CI 38.5–69.7) for PLR, and 844,000 (AUC 65.1%, 95% CI 50.2–79.9) for SII. We stratified the patients into high (≥3.3) and low (<3.3) NLR, high (≥170) and low (<170) PLR, and high (≥844,000) and low SII (<844,000) groups. Response evaluation was available in all patients. Six patients (9.7%) did not perform any HDCT cycles of carboplatin and etoposide because of progressive disease during mobilization chemotherapy and died after few months. Eight patients performed only one (*n* = 4, 6.5%) or two (*n* = 4, 6.5%) cycles of HDCT due to disease progression (*n* = 5, 8%) and toxicity (*n* = 3, 4.8%), two of them obtained SD and one had CR. The remaining 48 (77.4%) underwent three cycles of HDCT with PBPC. Twenty-six patients achieved CR (11 cCR, 10 pCR, and five sCR), and they are still alive to date. RPm– was achieved in 10 patients, RPm+ in seven patients, SD in three patients, and PD in two patients. Eighteen patients who underwent three cycles of carboplatin and etoposide died during follow up. NLR (*p* = 0.003), PLR (*p* = 0.006) as well as SII (*p* = 0.0001) were associated with overall response to HDCT (Table 2). Low NLR, PLR, and SII correlate with CR/PRm-. On the contrary, higher values of NLR, PLR and SII, are associated with PD. In addition, patients with very low/low/intermediate IPFSG risk as well as patients performing HDCT in second line have reported a significantly higher rate of CR/PRm- compared to high/very high risk (*p* = 0.005) and to patients receiving HDCT in third line or beyond third line (*p* = 0.008), respectively (Table 2).

**Table 2:** Correlation analysis between inflammation markers, prognostic IPFSG score, and previous lines of treatment with overall response.

	**Overall response**			***p***
	**CR, PRm-**	**PRm+, SD**	**PD**	
	***N* (%)**	***N* (%)**	***N* (%)**	
**NLR**				
<3.3	23 (76.7)	5 (16.7)	2 (6.6)	
≥3.3	14 (43.7)	7 (21.9)	11 (34.4)	0.003
**PLR**				
<170	22 (75.9)	5 (17.2)	2 (6.9)	
≥170	15 (45.5)	7 (21.2)	11 (33.3)	0.006
**SII**				
<844,000	26 (78.8)	6 (18.2)	1 (3.0)	
≥844,000	11 (37.9)	6 (20.7)	12 (41.4)	0.0001
**Previous lines of treatment**				
2	27 (71.1)	7 (18.4)	4 (10.5)	
≥3	10 (41.7)	5 (20.8)	9 (37.5)	0.008
**Prognostic IPFSG score**				
Low + intermediate risk	19 (79.2)	4 (16.7)	1 (4.1)	
High risk	18 (47.4)	8 (21.0)	12 (31.6)	0.005

*NLR, neutrophil-to-lymphocyte ratio; PLR, platelet-to-lymphocyte ratio; SII, systemic immune-inflammation index; IPFSG, International Prognostic Factor Study Group; CR, complete response; PRm–, markers negative partial response; PRm+, markers positive partial response; SD, stable disease; PD, progressive disease*.

The univariate Cox regression analysis has reported a significantly shorter PFS in patients with high NLR [hazard ratio (HR) 3.35 (95% CI 1.59–7.06); *p* = 0.001] and high SII [HR 2.84 (95% CI 1.41–5.70); *p* = 0.003] compared to patients with lower NLR and SII, respectively (Table 3). High NLR [HR 3.62 (95% CI 1.59–8.23); *p* = 0.002] and high SII [HR 2.87 (95%, 1.33–6.19); *p* = 0.0070 significantly correlate with shorter OS. In the univariate analysis, PLR was not significantly associated with OS and PFS (Table 3). Patients who received treatment beyond second line had a significant reduction in PFS [HR 2.35 (95% CI 1.19–4.63); *p* = 0.014] and OS [HR 2.40 (95% CI 1.15–5.01); *p* = 0.019] compared to patients treated in second line (Table 3). Then, we performed a multivariable analysis that included inflammatory markers (NLR, PLR, and SII), IPFSG risk group (very high/high vs. low/intermediate risk), age, and previous line of treatment (second vs. third or beyond third line). The multivariate analysis revealed that both NLR [HR 3.68 (95% CI 1.66–8.19); *p* = 0.001] and SII [HR 3.02 (95% CI 1.47–6.2); *p* = 0.003] remained independent predictors of PFS (Table 4). In multivariate analysis for OS, lower NLR [HR 4.49 (95% CI 1.86–10.82); *p* = 0.0008] and SII [HR 3.00 (95% CI 1.34–6.69); *p* = 0.007] correlate with longer OS (Table 4). In multivariate analysis, PLR was not a prognostic independent factor for OS [HR 1.51 (95% CI 0.68–3.37); *p* = 0.31] (Table 4).

**Table 3:** Univariate analysis of PFS and OS (median follow-up: 68 months, range: 4–110 months).

	**PFS**					**OS**				
	***N*, patients**	***N*, events**	**Median PFS (months) (95% CI)**	***p***	**HR (95% CI)**	***p***	***N*, events**	**Median OS (months) (95% CI)**	***p***	**HR (95% CI)**	***p***
Overall	62	34	12.3 (5.7–nr)	–	–	–	29	nr	–	–	–
**NLR**											
<3.3	30	10	nr		1.00		8	nr		1.00	
≥3.3	32	24	5.9 (4.3–10.0)	0.0008	3.35 (1.59–7.06)	0.001	21	14.2 (8.8–43.5)	0.001	3.62 (1.59–8.23)	0.002
**PLR**											
<170	29	13	nr		1.00		11	nr		1.00	
≥170	33	21	6.1 (4.5–nr)	0.082	1.83 (0.92–3.67)	0.087	18	43.5 (10.0–nr)	0.225	1.58 (0.75–3.36)	0.229
**SII**											
<844,000	33	13	nr		1.00		10	nr		1.00	
≥844,000	29	21	5.2 (3.6–11.9)	0.002	2.84 (1.41–5.70)	0.003	19	14.2 (9.6–nr)	0.005	2.87 (1.33–6.19)	0.007
**Previous lines of treatment**											
2	38	16	nr		1.00		14	nr		1.00	
≥3	24	18	6.1 (4.1–11.9)	0.011	2.35 (1.19–4.63)	0.014	15	14.9 (8.8–nr)	0.016	2.40 (1.15–5.01)	0.019

*NLR, neutrophil-to-lymphocyte ratio; PLR, platelet-to-lymphocyte ratio; SII, systemic immune-inflammation index; nr, not reached; PFS, progression-free survival; OS, overall survival; HR, hazard ratio; 95% CI, 95% confidence interval*.

**Table 4:** Multivariate analysis of PFS and OS (adjusted by age, previous lines of treatment, and prognostic score).

	**PFS**		**OS**	
	**HR (95% CI)**	***p***	**HR (95% CI)**	***p***
NLR (≥3.3 vs. <3.3)	3.68 (1.66–8.19)	0.001	4.49 (1.86–10.82)	0.0008
PLR (≥170 vs. <170)	2.18 (1.02–4.68)	0.045	1.51 (0.68–3.37)	0.314
SII (≥844,000 vs. <844,000)	3.02 (1.47–6.20)	0.003	3.00 (1.34–6.69)	0.007

*NLR, neutrophil-to-lymphocyte ratio; PLR, platelet-to-lymphocyte ratio; SII, systemic immune-inflammation index; PFS, progression-free survival; OS, overall survival; HR, hazard ratio; 95% CI, 95% confidence interval*.

## Discussion

Relapsed or refractory GCT patients represent a heterogeneous group of patients. In order to stratify this population, Lorch et al. (10) developed the IPFSG prognostic score based on six variables: primary site, first-line response, platinum-free interval, presence of bone, liver, or brain metastasis, and tumor markers (human chorionic gonadotropin and alpha-fetoprotein) level at baseline of salvage chemotherapy. Giving a score point to each category, the system is able to divide the patients into five prognostic groups: very low, low, intermediate, high, and very high. Two-year PFS changed from 75.1% in very low risk patients to 5.6% in very high-risk patients; therefore, the IPFSG prognostic score may help in clinical treatment decision. However, the high complexity of this population as well as the high treatment-related toxicity rates deserve the search for additional prognostic factors.

In recent decades, several studies investigate the link between inflammation and cancer: immune cells secrete cytokines and chemokines that promote tumor growth, angiogenesis, and metastasis development (18). Both neutrophils and platelets can induce tumor cell trans-endothelial migration and metastasis (19–21). On the contrary, lymphocytes can induce host immune response to cancer cells by inhibiting tumor cell proliferation and inducing cytotoxic cell death (17, 22). Since the identification of the relationship among inflammation cells, substances produced by inflammation, and tumor growth and microenvironment, the role of prognostic scores based on peripheral inflammation cells has been evaluated in several tumors (11–15).

The association of blood-based systemic inflammatory markers with the prognosis and the outcome of patients with GCT has been investigated in recent years.

In the preoperative setting, NLR could be useful to predict TNM staging in patients with testicular germ cell tumors (23). Therefore, a previous study did not find a correlation between NLR and cancer-specific survival and PFS in a small cohort of patients with GCT (24). Other studies evaluated the more recently developed marker SII and showed a worse prognosis of high SII in patients with metastatic GCT who are undergoing first-line chemotherapy (25, 26).

Based on the role of inflammation in tumor biology as well as the results of previous studies, we evaluated the correlation of inflammatory markers and the outcome of patients undergoing salvage therapy with HDCT. HDCT is an effective strategy in relapsed/refractory GCT, although it can be burdened with toxicity and consequently it should be performed in referring institutions (27, 28).

To the best of our knowledge, this is the first study that evaluates inflammation markers obtained from routine blood tests as an independent predictor of HDCT outcome. The present investigation reveals that SII and NLR can be predictors of PFS and OS. Moreover, NLR, SII, and PRL correlate with overall response to HDCT.

When we adjusted the analysis by IPFSG prognostic score and line of treatment, SII and NLR remained as independent predictors for OS and PFS, besides IPFSG prognostic score and line of treatment.

Our results indicated a slight, albeit not significant, benefit in PFS in those patients with a lower PLR value.

For the first time, our analysis suggests that inflammatory markers have the ability to improve the prediction of clinical outcome in the heterogeneous population of patients with relapsed/refractory GCT.

Associating IPFSG prognostic score to inflammation markers at baseline of HDCT may improve prognostic information and might help physicians to make more personalized treatment decisions.

Moreover, IPFSG prognostic score was not studied in patients treated beyond the second line of treatment. In these patients, SII and NLR can predict the oncological outcome.

The present study has some limitations related to the retrospective nature of data collection and to the apparently limited number of patients enrolled. Therefore, our analysis should be considered as explorative and should induce a multicenter harvest of a larger data in order to exactly determine the prognostic role of inflammatory markers in combination with the well-established IPFSG prognostic score.

In our analysis, blood-based systemic inflammatory markers have been evaluated at baseline of HDCT without following their trend during treatment because of the presence of many interfering factors during and after HDCT, mainly bone marrow toxicity and stem cell reinfusion.

In conclusion, SII and NLR can improve prognostic information in addition to IPFSG prognostic score for patients with metastatic GCT who are undergoing salvage chemotherapy.

## Data Availability Statement

The raw data supporting the conclusions of this article will be made available by the authors, without undue reservation.

## Ethics Statement

The studies involving human participants were reviewed and approved by IRST Ethical Committee. The patients/participants provided their written informed consent to participate in this study.

## Author Contributions

MC and UD contributed to the conception and design of this work. UD supervised the study. All authors contributed to the analysis and interpretation of literature, and writing, review, and/or revision of the manuscript. All authors read and approved the final manuscript.

## Conflict of Interest

UD has received personal fees for advisory board/consultancy from Astellas, Bayer, BMS, Ipsen, Janssen, Merck, Novartis, Pfizer, and Sanofi. GT has received personal fees for advisory board/consultancy from Novartis, Pfizer, Roche, and Italfarmaco. The remaining authors declare that the research was conducted in the absence of any commercial or financial relationships that could be construed as a potential conflict of interest.

## References

[1] Lorch A Beyer J Algaba F Bokemeyer C Cohn-Cedermark G Horwich A. How we treat germ cell cancers. Cancer. (2017) 123:2190–92. 10.1002/cncr.30751 28513824

[2] Fizazi K Oldenburg J Dunant A Chen I Salvioni R Hartmann JT. Assessing prognosis and optimizing treatment in patients with postchemotherapy viable nonseminomatous germ-cell tumors (NSGCT): results of the sCR2 international study. Ann Oncol. (2008) 19:259–64. 10.1093/annonc/mdm472 18042838

[3] Loehrer PJ Gonin R Nichols CR Weathers T Einhorn LH Vinblastine plus ifosfamide plus cisplatin as initial salvage therapy in recurrent germ cell tumor. J Clin Oncol. (1998) 16:2500–4. 10.1200/JCO.1998.16.7.2500 9667270

[4] Kondagunta GV Bacik J Donadio A Bajorin D Marion S Sheinfeld J. Combination of paclitaxel, ifosfamide, and cisplatin is an effective second-line therapy for patients with relapsed testicular germ cell tumors. J Clin Oncol. (2005) 23:6549–55. 10.1200/JCO.2005.19.638 16170162

[5] Bokemeyer C Kollmannsberger C Harstrick A Beyer J Gerl A Casper J. Treatment of patients with cisplatin-refractory testicular germ-cell cancer. Int J Cancer. (1999) 83:848–51. 10.1002/(sici)1097-0215(19991210)83:6<848::aid-ijc29>3.0.co;2-# 10597209

[6] De Giorgi U Rosti G Aieta M Testore F Burattini L Fornarini G. Phase II study of oxaliplatin and gemcitabine salvage chemotherapy in patients with cisplatin-refractory nonseminomatous germ cell tumor. Eur Urol. (2006) 50:1032–8. 10.1016/j.eururo.2006.05.011 16757095

[7] De Giorgi U Rosti G Slavin S Yaniv I Harousseau JL Ladenstein R. Parties salvage high-dose chemotherapy for children with extragonadal germ-cell tumors. Br J Cancer. (2005) 93:412–7. 10.1038/sj.bjc.6602724 16106248 PMC2361583

[8] De Giorgi U Demirer T Wandt H Taverna C Siegert W Bornhauser M. Second-line high-dose chemotherapy in patients with mediastinal and retroperitoneal primary non-seminomatous germ cell tumors: the EBMT experience. Ann Oncol. (2005) 16:146–51. 10.1093/annonc/mdi017 15598952

[9] Feldman DR Lorch A Kramar A Albany C Einhorn LH Giannatempo P. Brain metastases in patients with germ cell tumors: prognostic factors and treatment options–an analysis from the global germ cell cancer group. J Clin Oncol. (2016) 34:345–51. 10.1200/JCO.2015.62.7000 26460295 PMC5070579

[10] Lorch A Beyer J Bascoul-Mollevi C Kramar A Einhorn LH Necchi A. Prognostic factor in patients with metastatic germ cell tumors who experienced treatment failure with cisplatin-based first line chemotherapy. J Clin Oncol. (2010) 28:4906–11. 10.1200/JCO.2009.26.8128 20956623

[11] Rossi L Santoni M Crabb SJ Scarpi E Burattini L Chau C. High neutrophil-to-lymphocyte ratio persistent during first-line chemotherapy predicts poor clinical outcome in patients with advanced urothelial cancer. Ann Surg Oncol. (2015) 22:1377–84. 10.1245/s10434-014-4097-4 25234022

[12] Lolli C Basso U Derosa L Scarpi E Sava T Santoni M. Systemic immune-inflammation index predicts the clinical outcome in patients with metastatic renal cell cancer treated with sunitinib. Oncotarget. (2016) 7:54564–71. 10.18632/oncotarget.10515 27409344 PMC5342364

[13] Zheng J Cai J Li H Zeng K He L Fu H. Neutrophil to lymphocyte ratio and platelet to lymphocyte ratio as prognostic predictors for hepatocellular carcinoma patients with various treatments: a meta-analysis and systematic review. Cell Physiol Biochem. (2017) 44:967–81. 10.1159/000485396 29179180

[14] Passardi A Scarpi E Cavanna L Dall'Agata M Tassinari D Leo S. Inflammatory indexes as predictors of prognosis and bevacizumab efficacy in patients with metastatic colorectal cancer. Oncotarget. (2016) 7:33210–9. 10.18632/oncotarget.8901 27120807 PMC5078087

[15] Lolli C Caffo O Scarpi E Aieta M Conteduca V Maines F. Systemic immune-inflammation index predicts the clinical outcome in patients with mCRPC treated with abiraterone. Front Pharmacol. (2016) 7:376. 10.3389/fphar.2016.00376 27790145 PMC5062111

[16] Feldman DR Sheinfeld J Bajorin DF Fischer P Turkula S Ishill N. TI-CE high-dose chemotherapy for patients with previously treated germ cell tumors: results and prognostic factor analysis. J Clin Oncol. (2010) 28:1706–13. 10.1200/JCO.2009.25.1561 20194867 PMC3651604

[17] De Giorgi U Mego M Scarpi E Giuliano M Giordano A Reuben JM. Relationship between lymphocytopenia and circulating tumor cells as prognostic factors for overall survival in metastatic breast cancer. Clin Breast Cancer. (2012) 12:264–9. 10.1016/j.clbc.2012.04.004 22591634

[18] Mantovani A Allavena P Sica A Balkwill F. Cancer-related inflammation. Nature. (2008) 454:436–44. 10.1038/nature07205 18650914

[19] Schumacher D Strilic B Sivaraj KK Wettschureck N Offermanns S. Platelet-derived nucleotides promote tumorcell transendothelial migration and metastasis via P2Y2 receptor. Cancer Cell. (2013) 24:130–7. 10.1016/j.ccr.2013.05.008 23810565

[20] Labelle M Begum S Hynes RO. Direct signaling between platelets and cancer cells induces epithelial-mesenchymallike transition and promote metastasis. Cancer Cell. (2011) 20:576–90. 10.1016/j.ccr.2011.09.009 22094253 PMC3487108

[21] Cools-Lartigue J Spicer J McDonald B Gowing S Chow S Giannias B. Neutrophyl extracellular traps sequester circulating tumor cells and promote metastasis. J Clin Invest. (2013) 123:3446–58. 10.1172/JCI67484 23863628 PMC3726160

[22] Minami T Minami T Shimizu N Yamamoto Y De Velasco M Nozawa M. Identification of programmed death ligand 1-derived peptides capable of inducing cancerreactive cytotoxic T lymphocytes from HLA-A24+ patients with renal cell carcinoma. J Immunother. (2015) 38:285–91. 10.1097/CJI.0000000000000090 26261892

[23] Jankovich M Jankovichova T Ondrus D Breza J. Neutrophil-to-lymphocyte ratio as a predictor of preoperative tumor staging in testicular germ cell tumors. Bratisl Med J. (2017) 118:510–2. 10.4149/BLL_2017_098 29061055

[24] Bolat D Aydogdu Ö Polat S Yarimoglu S Bozkurt IH Yonguç T. Predictive value of preoperative neutrophil-to-lymphocyte ratio on the prognosis of germ cell testicular tumors. Turk J Urol. (2017) 43:55–61. 10.5152/tud.2016.38924 28270952 PMC5330269

[25] Chovanec M Cierna Z Miskovska Machalekova K Kalavska K Rejlekova K. Systemic immune-inflammation index in germ-cell tumours. Br J Cancer. (2018) 118:831–8. 10.1038/bjc.2017.460 29485980 PMC5877428

[26] Fankhauser CD Sander S Roth L Oross O Eberli D Sulser T. Systemic inflammatory markers have independent prognostic value in patients with metastatic testicular germ cell tumours undergoing first-line chemotherapy. Br J Cancer. (2018) 118:825–30. 10.1038/bjc.2017.467 29485982 PMC5877429

[27] Adra N Abonour R Althouse S Albany C Hanna NH Einhorn LH. High-dose chemotherapy and autologous peripheral-blood stem-cell transplantation for relapsed metastatic germ cell tumors: the Indiana University experience. J Clin Oncol. (2017) 35:1096–102. 10.1200/JCO.2016.69.5395 27870561 PMC5455354

[28] Hanna NH Einhorn LH. Testicular cancer—discoveries and updates. N Engl J Med. (2014) 371:2005–16. 10.1056/NEJMra1407550 25409373

